# Prevalence and incidence of neuropsychiatric disorders in post hospitalized COVID-19 patients in South America: a systematic review and meta-analysis

**DOI:** 10.3389/fpsyt.2023.1163989

**Published:** 2023-11-01

**Authors:** Francisco Perea-Flórez, Nair Javier-Murillo, André Lapeyre-Rivera, Bryan Gamonal, Miguel Cabanillas-Lazo, Victor Velásquez-Rimachi, Carlos Alva-Diaz

**Affiliations:** ^1^Facultad de Medicina Humana, Universidad de Piura, Lima, Peru; ^2^Sociedad Científica de la Universidad de Piura, Lima, Peru; ^3^Red de Eficacia Clínica y Sanitaria, REDECS, Lima, Peru; ^4^Facultad de Medicina, Universidad Nacional Mayor de San Marcos, Lima, Peru; ^5^Sociedad Científica de San Fernando, Lima, Peru; ^6^Grupo de Investigación en Neurociencia, Efectividad Clínica y Salud Pública, Universidad Científica del Sur, Lima, Peru; ^7^Facultad de Medicina de la Universidad Señor de Sipán, Chiclayo, Peru; ^8^Departamento de Medicina y Oficina de Apoyo a la Docencia e Investigación (OADI), Servicio de Neurología, Hospital Daniel Alcides Carrión, Callao, Peru

**Keywords:** neuropsychiatric, long COVID-19, South America, post-hospitalized, post-COVID

## Abstract

**Introduction:**

There are multiple reports of neuropsychiatric disorders (NDs) such as stress, depression, post-traumatic stress disorder (PTSD), or anxiety, in patients who have survived the acute phase of COVID-19, being even more frequent in people who were hospitalized with moderate or severe disease. South America (SA) was one of the most affected continents during this time due to its health, social, political and economic context. We aimed to determine the prevalence and incidence of NDs in patients following hospitalization for COVID-19 in SA.

**Materials and methods:**

We searched in PubMed, Embase, Scopus, Web of Science, LILACS, SciELO, and Google Scholar databases up to October 2022. We performed proportion meta-analysis with a random-effect model and Freeman-Tukey Double Arcsine transformation using the STATA 16.1 program. Finally, we evaluated heterogeneity by subgroup analysis and certainty of evidence with the GRADE approach.

**Results:**

We included eight studies from four countries. We only pooled six studies with prevalence measures. The estimated prevalence of all NDs was 31.48% (two-studies, 95%CI: 25.82–37.43). Depression, anxiety, insomnia, PTSD, and memory alterations had a pooled prevalence of 16.23% (three-studies, 95%CI: 7.18–27.93, I2: 94.22), 18.72% (three-studies, 95%CI: 11.65–26.97, I2: 87.56), 43.07% (three-studies, 95%CI: 32.77–53.37, I2: 92.61), 31.78% (three-studies, 95%CI: 14.33–52.40, I2: 97.96), and 38.24% (two-studies, 95%CI: 35.5–40.97), respectively. The evidence included was deemed as moderate to high certainty.

**Conclusion:**

We suggest that NDs should be prioritized in research and care in South America with public policies that can support their identification and prompt management to improve the quality of life of patients. More studies are needed to adequately study the prevalence of NDs in South America, their associated factors, and evaluate the causes of heterogeneity.

**Systematic review registration:**

https://doi.org/10.6084/m9.figshare.21901041.v1.

## 1. Introduction

The COVID-19 pandemic has affected a vast number of individuals globally, with over 750 million confirmed cases reported as of February 2023 (1), causing from asymptomatic infection to severe illness and death. South America (SA) has been one of the regions most affected by the pandemic, presenting 9% of cases worldwide and up to 20% of the total deaths caused by the virus (2).

Like many diseases, sequelae may be present after having overcome the virus, regardless of the severity of the acute process (3). The World Health Organization defines “post-COVID,” “post-acute sequelae of COVID-19,” or “long COVID” as the maintenance or development of new clinical manifestations 3 months after acute infection by SARS-CoV-2, lasting at least 2 months and that cannot be explained by another cause (4). This situation was not initially evident due to the focus on controlling the spread and managing acute symptoms during the initial phases of the pandemic. Compromised physical and mental health aspects have been reported, the most common being fatigue, dyspnea, and cognitive deterioration, although more than 200 associated symptoms have been described to date, especially in hospitalized compared to non-hospitalized patients (4–6). It is a well-established fact that post-COVID syndrome tends to be more prevalent among patients who have endured severe cases of COVID-19. The term “post-hospitalized patients” pertains to individuals who have undergone moderate to severe manifestations of the disease, classifying them as a population at high risk for the manifestation of post-COVID syndrome (7). COVID-19 has the capacity to induce widespread inflammation within the body, including the brain, thereby potentially giving rise to an array of neurological complications, including psychiatric manifestations and it may be involved in the onset of depressive and anxious symptoms in post hospitalized COVID-19 patients (8). Those patients who have undergone hospitalization due to COVID-19 could have encountered an elevated degree of emotional stress resulting from their confinement in the hospital environment and the ailment itself, thus potentially contributing to the emergence of symptoms indicative of anxiety and depression. In post-hospitalized COVID-19 patients, there is a higher risk of developing anxiety and depression disorders compared to the general population (9).

Neuropsychiatric symptoms, such anxiety, depression, post-traumatic stress disorder (PTSD), sleep disorders, and among others, have been reported as part of long COVID. Different reviews have described the existence of different neuropsychiatric symptoms, but there is no consensus on which is the most frequent (10–14), as this varies depending on the different characteristics of the participants and studies.

Some systematic reviews already estimated the high frequency of these neuropsychiatric disorders in post-COVID patients, but none has focused specifically on SA, despite being a continent heavily impacted by this pandemic. Therefore, our main objective was to determine the prevalence and incidence of neuropsychiatric disorders in patients following hospitalization for COVID-19 in SA.

## 2. Methods

This systematic review was reported according to the Preferred Reporting Items for Systematic Reviews and Meta-Analyses (PRISMA) (15). The study protocol was registered in Figshare (10.6084/m9.figshare.21901041).

### 2.1. Terms and definitions

For the concept of neuropsychiatric sequelae, we have adopted the definition provided by the WHO, mentioned above (4). In the studies, SARS-CoV-2 infection was defined based on the PCR result or bronchoalveolar lavage. The neuropsychiatric symptoms considered in the search included anxiety, depression, post-traumatic stress disorder (PTSD), sleep disorders, concentration difficulties, phobias, obsessive-compulsive disorder, hallucinations, headaches, hyposmia, dysgeusia, dizziness, delusions, visual disturbances, auditory disorders, paresthesia, weakness, gait abnormalities, and consciousness disorders.

We have considered the reports of those articles that measured these symptoms using validated instruments or self-reports and that followed standard diagnostic criteria such as the Diagnostic and Statistical Manual of Mental Disorders (DSM) or the International Classification of Diseases (ICD).

### 2.2. Data sources

We searched PubMed, Embase, Scopus, Web of Science, LILACS, SciELO, and Google Scholar up to October 2022. The search strategy for each database was developed by the senior authors (Supplementary material). There were no restrictions on language or publication date.

### 2.3. Eligibility criteria

The eligibility criteria included observational, cross-sectional or longitudinal studies of post-hospitalized COVID-19 patients in whom the frequency of neuropsychiatric disorders was estimated. We excluded letters to the editor, case-reports, case series, experimental studies, conferences, abstract and review studies, including systematic reviews and epidemiological studies (prevalence or incidence).

### 2.4. Study selection and data extraction

The electronic search results were imported into Endnote X9 to delete duplicate records. Then, we exported all the articles to Rayyan^1^ to be screened independently using the title and abstract by two reviewers, and any discrepancies were resolved by consensus and consideration of the opinion of a third reviewer. The reviewers assessed the inclusion criteria independently by reading the full texts of potentially relevant studies, and discrepancies were resolved according to consensus.

The following data were extracted from the individual studies: general information on the article (title, first author, country of origin, and year of publication), methodological characteristics (study design, year of data collection, and subject selection), relevant information for data analysis (total number of post-hospitalized subjects, age and sex of the participants, comorbidities, admission to intensive care unit, etc.), and the prevalence/incidence of neuropsychiatric disorders. After data extraction, all the data were coded for analysis.

### 2.5. Risk of bias assessment

Bias was assessed independently by three investigators (M.C, A.L, and B.G) using the Joanna Briggs Institute (JBI) critical appraisal tools for incidence and prevalence studies (16). Each item has sub items to which a star-based score is assigned. The risk of bias of individual studies was determined with the following cutoffs: low risk of bias if 70% of answers scored yes, moderate risk if 50–69% questions scored yes, and high risk of bias if yes scores were below 50%.

### 2.6. Statistical analysis

Quantitative analysis through a meta-analysis was performed using a random-effects model. We used the Freeman–Tukey double arcsine transformation to stabilize the proportion variances (17). Variance among studies (*τ*^2^) was estimated using the DerSimonian–Laird estimator (18). Prevalence with the 95% confidence intervals (95% CI) were pooled and expressed as neuropsychiatric cases per 100 post-hospitalized COVID-19 patients, using a binomial model (metaprop command in Stata) (19). Heterogeneity among studies was assessed using the I2 statistic.

### 2.7. Certainty of evidence

Two authors independently assessed the certainty of our pooled results by applying the Grading of Recommendation, Assessment, Development, and Evaluation (GRADE) system (20). This assessment is based on five domains: study limitations (risk of bias of the studies included), imprecision (sample size and CI), indirectness (generalizability), inconsistency (heterogeneity), and publication bias as stated in the GRADE handbook. We adapted the assessment to prevalence estimates. The certainty of the evidence was characterized as high, moderate, low, or very low.

## 3. Results

### 3.1. Search results

**Figure 1:**
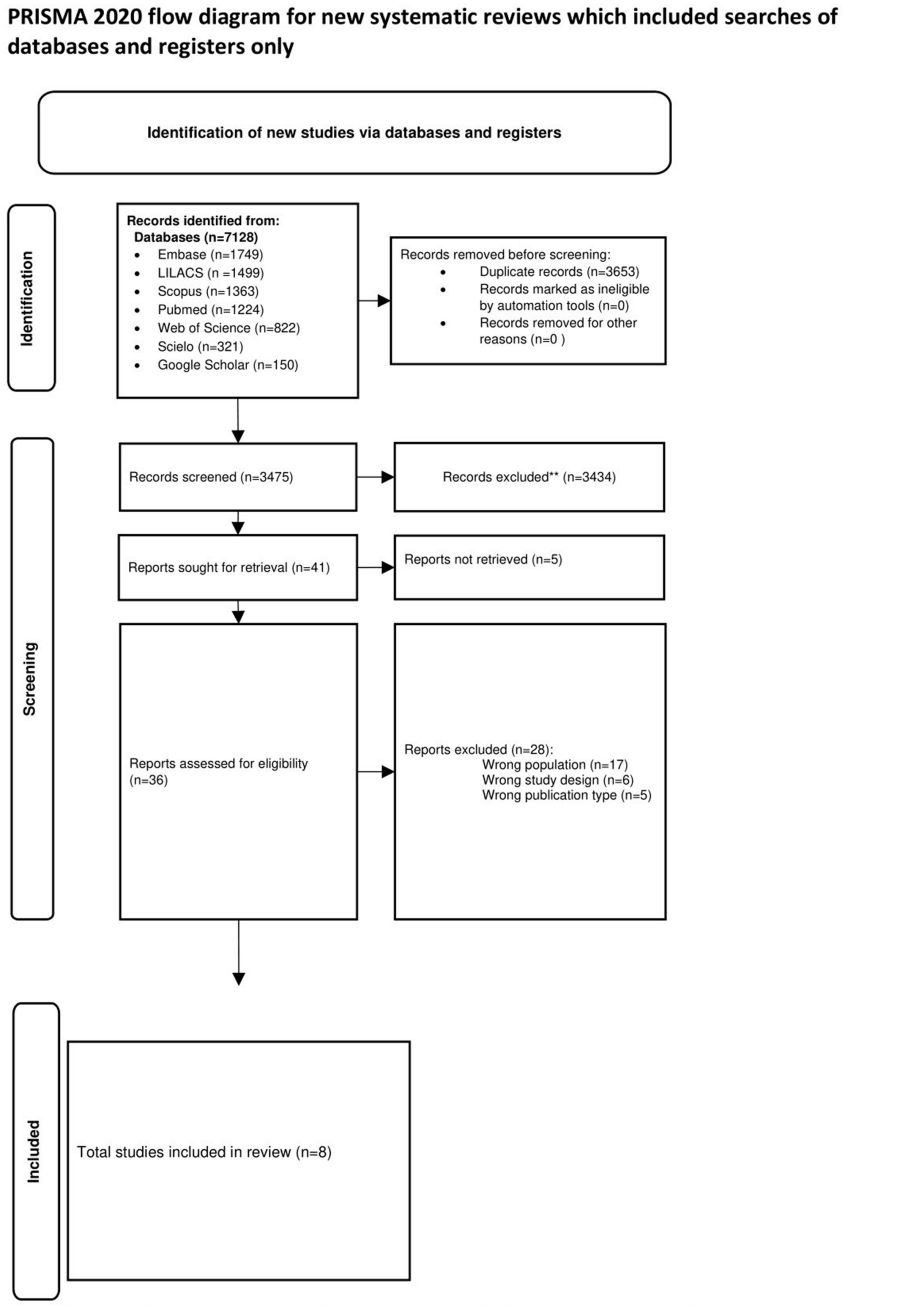
Flow chart for the selection of included studies.

We identified 7,128 titles from the database search. A total of 36 full-text studies were read and assessed. We included eight studies for final analysis. The detailed list of excluded studies can be found in the Supplementary material. Figure 1 shows a flow chart to illustrate the process of article selection and inclusion in the study.

### 3.2. Characteristics of included studies

Of the eight studies included, six were cross-sectional and two were longitudinal. The total number of participants was 2,635 individuals (sample size ranging from 42 to 801, median = 188.5), 1,408 (53.43%) of whom were males. The mean age ranged from 48.9 to 66.9 years, although age was not specified in the study by Rioja et al. (21). Most of the studies were conducted in Brazil (*n* = 4), followed by Peru (*n* = 2), Chile (*n* = 1), and Colombia (*n* = 1). One study only included intensive care unit patients (22). Only two of the longitudinal studies reported follow-up time and the mean was 7.5 months (23, 24). Six of the studies evaluated anxiety and/or depression, one evaluated only post-traumatic stress (PTSD) disorder, and one did not specify the evaluation. A summary of the methodological characteristics of the studies can be found in Table 1.

**Table 1:** Characteristics of the studies on neuropsychiatric disorders included in the review by prevalence and incidence in South America.

Author, year	Country	Design	Sample size	Age (mean, years)	Sex (*n*)	Follow-up time, strategy	Prevalence/Incidence	Type of patient	ND evaluated	Data year	Funding
Costa (24)	Brazil	Longitudinal	251	53.6	F: 101	3 months, telephone	Incidence	Hospitalized	Anxiety/depression	2020	Self-funded
					M: 150						
Carvalho (25)	Brazil	Cross-sectional	749	55	F: 352	6 months, in-person^*^	Prevalence	Hospitalized	Depression, anxiety, disturbance of concentration, insomnia, PTSD, headache, hyposmia, dysgeusia, memory disturbance, dizziness and disorientation, hearing impairment, paresthesia, weakness, gait disturbance, and altered consciousness	2020	Reported
					M: 397						
Damiano (26)	Brazil	Cross-sectional	425	55.7	F: 206	6–9 months, in-person*	Prevalence	Hospitalized	Depression, anxiety, phobia, OCD, PTSD, hallucinations, memory disturbance, and delirium	2020–2021	Reported
					M: 219						
Rizzo (27)	Brazil	Cross-sectional	801	55.35	F: 380	3–11 months, telephone and in-person*	Prevalence	Hospitalized	Anxiety/depression, insomnia	2020–2021	Reported
					M: 421						
Henríquez (28)	Chile	Cross-sectional	42	48.9	F: 16	4 months, in-person*	Prevalence	Hospitalized	Depression, anxiety, insomnia, and daytime sleepiness	2020	Not Reported
					M: 26						
Rojas Cárdenas (22)	Colombia	Cross-sectional	122	66.9	F: 46	No follow-up, medical record	Prevalence	ICU	Not specified	2020–2021	Not Reported
					M: 76						
Huarcaya (23)	Perú	Longitudinal	119	55	F: 55	3 and 12 months, telephone calls	Incidence	Hospitalized	Depression, anxiety	2020–2021	Reported
					M: 64						
Rioja (21)	Perú	Cross-sectional	126	-	F: 71	NR, telephone^*^	Prevalence	Hospitalized	PTSD	2021	Not Reported
					M: 55						

^*^The patient was followed up, but there was no baseline measurement of symptoms. NR, Not reported; ICU, Intensive care unit; PTSD, Post-traumatic stress disorder; and ND, neuropsychiatric disorder.

### 3.3. Risk of bias of the studies included

Six studies were assessed by the JBI for prevalence studies and two using the same tool for incidence. The risk of bias range of the included studies of neuropsychiatric disorders was from “low” to “moderate.” Six studies were deemed to have low risk of bias while two studies were considered to have moderate risk of bias. The percentage of “yes” in the tool items for prevalence studies in the low-risk studies was greater than or equal to 70%, being less than 70% but greater than 50% for moderate risk studies. A summary of the quality assessment of the studies can be found in Table 2.

**Table 2:** Quality assessment of prevalence (A) and incidence studies **(B)**.

A. Prevalence													
Authors	Q1	Q2	Q3	Q4	Q5	Q6	Q7	Q8	Q9	%Yes			Risk
Rojas Cardenas et al (22).	✓	✓	✓	✓	✓	✓	✓	✓	✓	100			Low
Ferreira et al (25).	—	—	✓	✓	✓	✓	✓	✓	—	66,6			Moderate
Rioja (21).	✓	—	—	—	✓	✓	✓	✓	✓	66,6			Moderate
Battistella et al. (27).	✓	—	✓	✓	✓	—	✓	✓	✓	77,7			Low
Damiano et al (26).	✓	✓	✓	✓	✓	U	✓	✓	✓	88,8			Low
Henríquez-Beltrán et al (28).	✓	✓	✓	✓	✓	✓	✓	✓	✓	100			Low
B. Incidence													
Authors	Q1	Q2	Q3	Q4	Q5	Q6	Q7	Q8	Q9	Q10	Q11	%Yes	Risk
Todt et al (24).	✓	✓	✓	✓	✓	✓	—	—	✓	✓	✓	81.8	Low
Huarcaya-Victoria et al (23).	✓	✓	—	✓	✓	✓	—	✓	—	✓	✓	72.7	Low

Q1: Was the sample frame appropriate to address the target population? Q2: Were study participants sampled in an appropriate way? Q3: Was the sample size adequate? Q4: Were the study subjects and the setting described in detail? Q5: Was the data analysis conducted with sufficient coverage of the identified sample? Q6: Were valid methods used for the identification of the condition? Q7: Was the condition measured in a standard, reliable way for all participants? Q8: Was there appropriate statistical analysis? Q9: Was the response rate adequate, and if not, was the low response rate managed appropriately? ✓, Yes; −, No; U, Unclear; and N/A, Not/Applicable.

### 3.4. Qualitative analysis

#### 3.4.1. Prevalence

One study described the overall prevalence of neuropsychiatric disorders (Rojas et al., *n* = 17 and prevalence of 13.93%) (22). In regard to the six prevalence studies, only one study reported depression and anxiety as a single variable (Rizzo et al., *n* = 467 and a prevalence of 58.3%) (27), while the other studies reported depression and anxiety separately, with prevalence ranging from 8–26.19% to 14.12–23.63%, respectively. With respect of insomnia and PTSD, the prevalence was 32.31–66.67% and 13.65–51.59%, respectively. Finally, only Carvalho et al. reported the prevalence of concentration (27.77%) and memory impairment (31.91%) (25).

#### 3.4.2. Incidence

Regarding incidence studies, none reported the overall prevalence of neuropsychiatric disorders. Costa et al. described depression and anxiety as a single variable with an incidence of 16.33%, while Huarcaya et al. reported depression and anxiety with an incidence of 36.97 and 31.09%, respectively (23, 24).

### 3.5. Quantitative analysis

**Figure 2:**
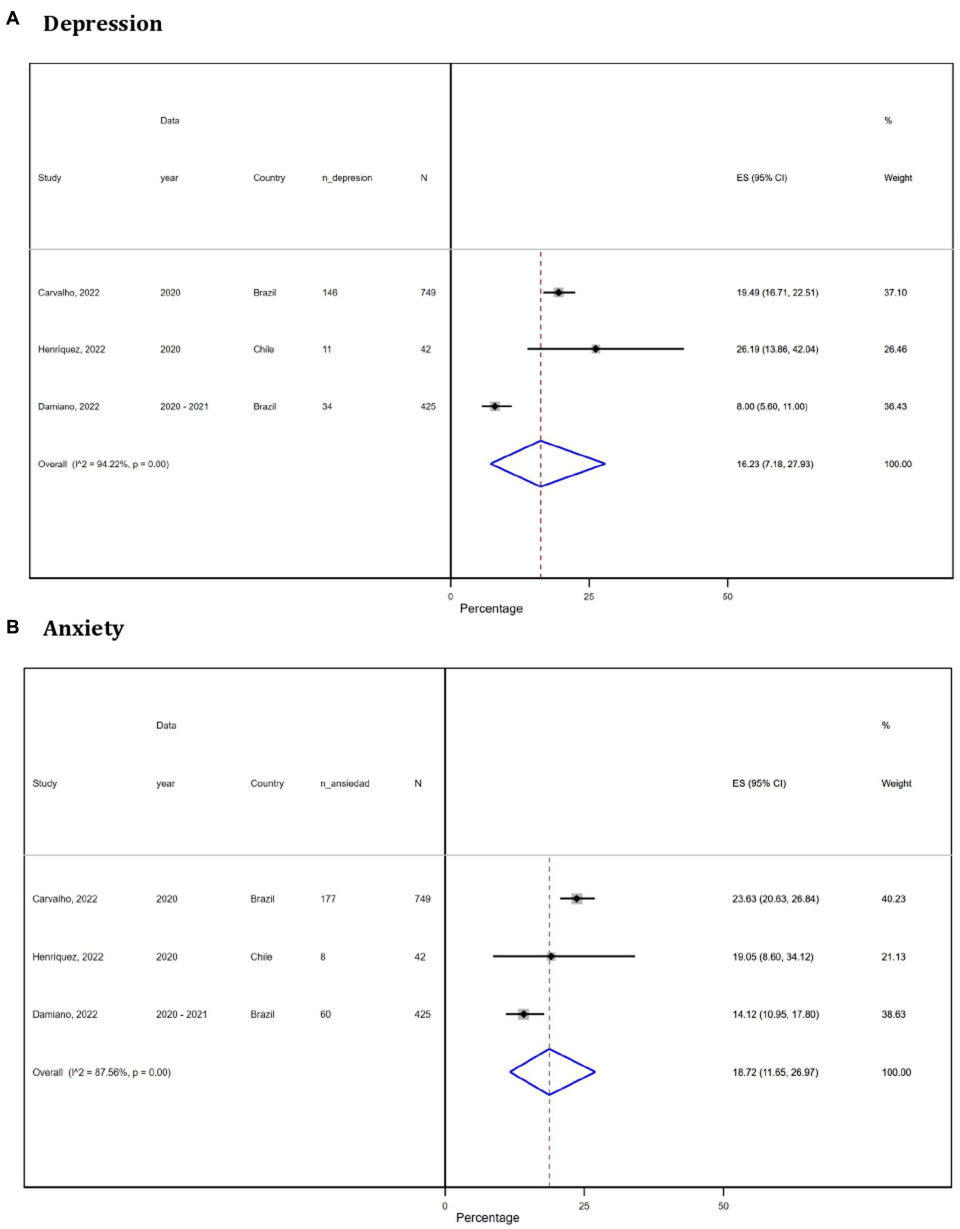

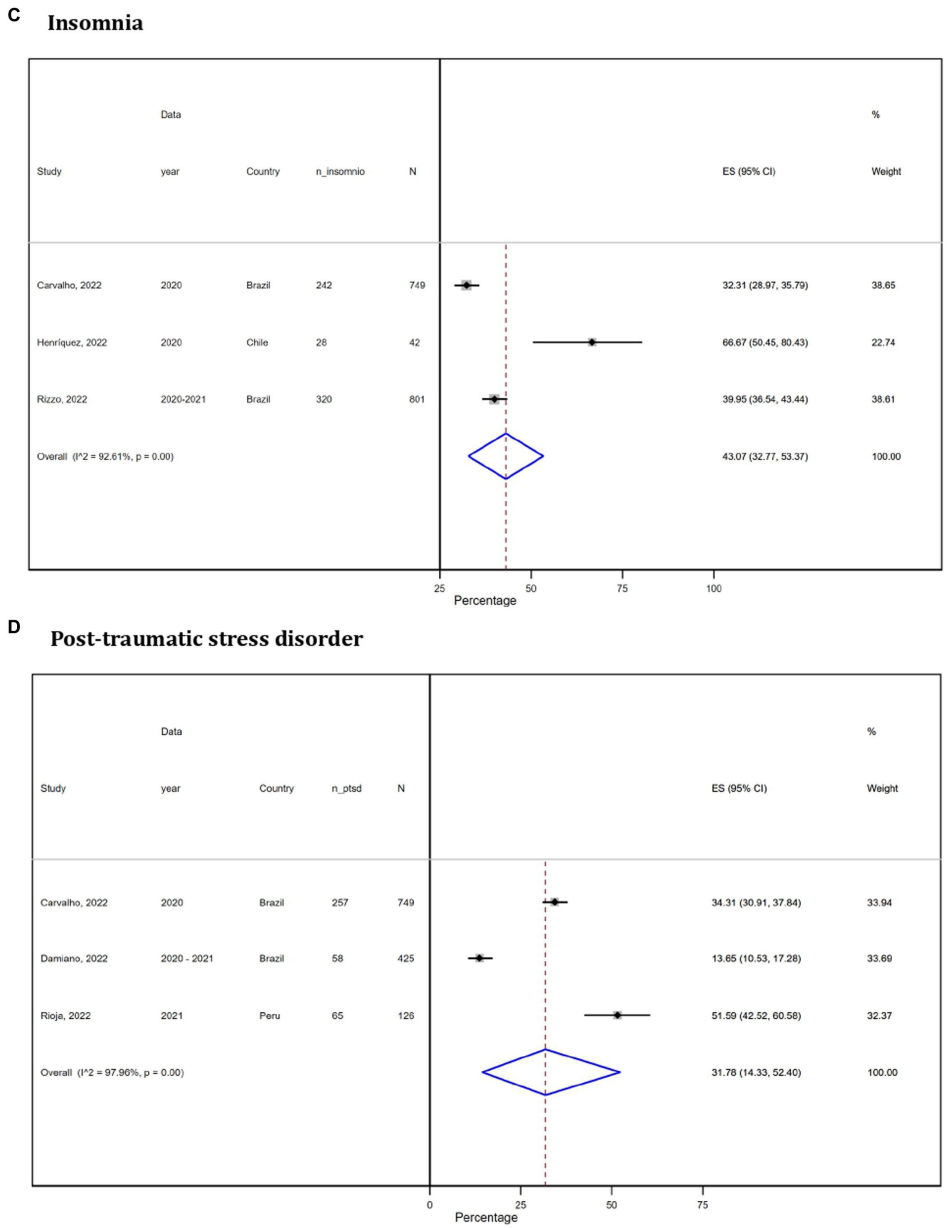

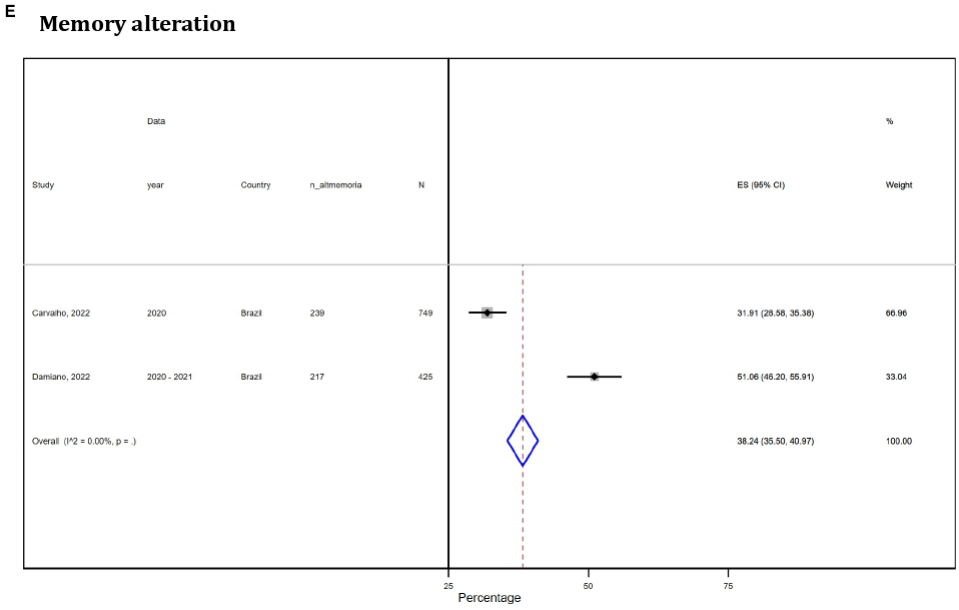
Forest plot (random-effects model) of meta-analysis on the prevalence of neuropsychiatric disorders in South America: depression **(A)**, anxiety **(B)**, insomnia **(C)**, post-traumatic stress disorder **(D)**, and memory alteration **(E)**.

Most of the articles specified the prevalence of the symptoms independently. The data were insufficient for incidence analysis as one study (24) reported anxiety and depression together, while another (23) reported them separately. Our analysis revealed the following pooled prevalence in SA: depression (16.23%, *n* = 3 studies, 95% CI: 7.18–27.93, I2: 94.22%), anxiety (18.72%, *n* = 3 studies, 95% CI: 11.65–26.97, I2: 87.56%), insomnia (43.07%, *n* = 3 studies, 95% CI: 32.77–53.37, I2: 92.61%), PTSD (31.78%, *n* = 3 studies, 95% CI: 14.33–52.40, I2: 97.96%), and memory alterations (38.24%, *n* = 3 studies, 95% CI: 35.50–40.97, I2: 0%; Figure 2).

### 3.6. Subgroup analysis

For each neuropsychiatric disorder, we stratified the studies according to country, gross national income *per capita*, the Human Development Index and altitude. There was insufficient data to perform an analysis by type of patient. The two studies (25, 26) that provided this information were from Brazil and presented a prevalence of depression (14.85%, 95% CI: 12.87–16.9), anxiety (19.97%, 95% CI: 17.73–22.3), insomnia (35.98%, 95% CI: 33.7–38.46), and PTSD (26.11%, 95% CI: 26.63–28.6).

### 3.7. Sensitivity analysis

The sensitivity analysis showed that the prevalence of neuropsychiatric disorders was higher in studies with moderate risk of bias for depression (19.49%, 95% CI: 16.71–22.5), anxiety (23.63%, 95% CI: 20.63–26.84), and PTSD (36.69%, 95% CI: 33.52–39.9) and in longitudinal studies on PTSD (34.31%, 95% CI: 30.91–37.84). While in low risk of bias studies with a cross-sectional design, the prevalence of neuropsychiatric disorders was higher for insomnia 41.38% (95% CI: 38.08–44.68) and 66.67% (95% CI: 50.45–80.43), respectively (see Table 3).

**Table 3:** Sensitivity analysis of meta-analysis about neuropsychiatric disorders prevalence in South America.

	Depression					Anxiety					Insomnia					Post-traumatic stress disorder				
	*N*	Prev.	95%CI	% weight	I2	*N*	Prev.	95%CI	% weight	I2	*N*	Prev.	95%CI	% weight	I2	*N*	Prev.	95%CI	% weight	I2
Risk of bias																				
BLow Bias	2	8.88	6.38–11.73	62.9	.	2	14.22	11.12–17.61	59.77	.	2	41.38	38.08–44.68	61.35	.	1	13.65	10.53–17.28	33.69	.
Moderate bias	1	19.49	16.71–22.51	37.1	.	1	23.63	20.63–26.84	40.23	.	1	32.31	28.97–35.79	38.65	.	2	36.69	33.52–39.93	66.31	.
Study design																				
Cross-sectional	2	8.88	6.38–11.73	62.9	.	2	14.22	11.12–17.61	59.77	.	1	66.67	50.45–80.43	22.74	.	2	20.87	17.56–24.38	66.06	.
Longitudinal	1	19.49	16.71–22.51	37.1	.	1	23.63	20.63–26.84	40.23	.	2	36.08	33.7–38.46	77.26	.	1	34.31	30.91–37.84	33.94	.
Sample number																				
<300	1	26.19	13.86–42.04	26.46	.	1	19.05	8.6–34.12	21.13	.	1	66.67	50.45–80.43	22.74	.	1	51.59	42.52–60.58	32.37	.
≥300	2	14.85	12.87–16.95	73.54	.	2	19.97	17.73–22.31	78.87	.	2	36.08	33.7–38.46	77.26	.	2	26.11	26.63–28.66	67.63	.

Prev., Prevalence.

### 3.8. Certainty of evidence

Initially there was a high certainty of evidence because all the studies included in meta-analyses were cross-sectional in in-hospital patients. For the prevalence of insomnia, we obtained a high certainty despite high heterogeneity since we evaluated a diverse region with expected heterogeneity, and thus, we decided not to downgrade. Moderate certainty of evidence was obtained for the prevalence of all neuropsychiatric disorders, depression, anxiety, post-traumatic stress, and memory alteration, due to most of the participants belonged to studies with a moderate risk of bias (Table 4).

**Table 4:** Quality of the body of evidence according to GRADE: summary of findings.

Outcomes	No. of participants (studies)	Certainty of the evidence (GRADE)	Anticipated absolute effects	
			Frequency pooled, %	95% CI
Prevalence of all neuropsychiatric sequelae per 100 patients	248 (2)	⨁⨁⨁◯ Moderate^a,b^	31.48	25.82–37.43
Prevalence of depression per 100 patients	1,216 (3)	⨁⨁⨁◯ Moderate^a,b^	16.23	7.18–27.93
Prevalence of anxiety per 100 patients	1,216 (3)	⨁⨁⨁◯ Moderate^a,b^	18.72	11.65–26.97
Prevalence of insomnia per 100 patients	1,592 (3)	⨁⨁⨁⨁ High^b^	43.07	32.77–53.37
Prevalence of post-traumatic stress disorder per 100 patients	1,300 (3)	⨁⨁⨁◯ Moderate^a,b^	31.78	14.33–52.40
Prevalence of memory alteration per 100 patients	1,174 (2)	⨁⨁⨁◯ Moderate^a,b^	38.24	35.50–40.97
CI: confidence interval				
GRADE Working Group grades of evidence				
High certainty: We are very confident that the true effect lies close to that of the estimate of the effect.				
Moderate certainty: We are moderately confident in the effect estimate; the true effect is likely to be close to the estimate of the effect, but there is a possibility that it is substantially different.				
Low certainty: Our confidence in the effect estimate is limited; the true effect may be substantially different from the estimate of the effect.				
Very low certainty: We have very little confidence in the effect estimate; the true effect is likely to be substantially different from the estimate of the effect.				

^a^Most of the participants belong to studies with moderate risk of bias. ^b^High inconsistency was detected. The calculated I2 was > 60%. We expected high heterogeneity, because we are evaluating a diverse region; thus, we decided not to downgrade.

## 4. Discussion

In this systematic review and meta-analysis, we found that the most prevalent neuropsychiatric disorders were insomnia (43.07%) followed by memory alterations (38.24%), PTSD (31.78%), anxiety (18.72%), and depression (16.23%).

In summary, we identified and included eight studies that provided information on neuropsychiatric disorders in post-hospitalized COVID-19 patients. The studies were performed in Brazil (4), Peru (2), Colombia (1), and Chile (1). Only one study reported the overall prevalence of neuropsychiatric disorders (Rojas et al., *n* = 17 and prevalence of 13.93%).

The finding of insomnia as the most prevalent neuropsychiatric disorder is in line with international literature, which describes insomnia and sleep disorders as among the most frequent manifestations of long-COVID (10). In the systematic review by Zeng et al. on recovered COVID-19 patients, the prevalence of sleep disorders was 13.5% (95% CI 8.7–19.2) (12), which was lower than the prevalence found in our study. This can be explained by the subgroup analysis carried out in the study by these authors, which showed a higher prevalence of sleep disorders in severe or critical patients compared to asymptomatic patients (12). The sleep disturbances could be due to various causes, some linked to the stress related to the infection itself, misinformation in the media, and the context of the pandemic. However, in post-hospitalized individuals, evidence suggests that it could be due to the release of proinflammatory cytokines from activation of the immune system (29), as well as respiratory sequelae following acute COVID infection (30).

Regarding memory impairment, we report a prevalence of 38.24%, which includes brain fog, loss of focus, and memory deterioration, which is common and has a negative impact on the patients’ lives. This prevalence appears higher than what has been reported in some other studies, where objective cognitive impairment has been reported as one of the most common manifestations of long-COVID, with a prevalence of 20.2% (95% CI, 10.3–35.7%) in survivors of COVID-19 without evidence of differential prevalence based on hospitalization status (25).

The prevalence of post-traumatic stress disorder (PTSD) in long-COVID has been reported to be between 10.5 and 37.2%, which is consistent with our estimate of 31.78% (25). The prevalence of anxiety and depression were 18.72% (11.65–26.97%) and 16.23% (7.18–27.93%), respectively. These findings confirm that depression and anxiety are serious problems in patients with prolonged COVID, as has been shown in previous studies (8). Zeng et al. reported a prevalence of anxiety of 20.7 and 12.9% in mild/moderate and severe patients in hospital, respectively (10). Similarly, a systematic review of neuropsychological and psychiatric sequelae of COVID-19 found that between 10.0 and 19.0% of previously hospitalized patients reported moderate to severe depression (31). In the study by Zeng et al., a prevalence of depression of 16.8 and 10.2% was reported in mild/moderate and severe patients in hospital, respectively (10).

While it is improbable to definitively ascertain the reasons for the higher frequency of neuropsychiatric sequelae in South America (SA), several factors that are likely associated with these differences can be identified.

### 4.1. High percentage of informal economy

Informal labor encompasses economic activities carried out by workers and/or economic units that, in either legal or practical terms, lack or have insufficient coverage by formal arrangements. The absence of a stable monthly income compels these workers to rely on daily production, while the absence of occupational health insurance hinders access to medical care. Articles within this systematic review stem from Brazil, Peru, Colombia, and Chile, countries that exhibited non-agricultural informal economy percentages of 44.9, 59.9, 57.3, and 27.8% respectively, in 2019 (32). This reality underscores that a significant proportion of SA’s population is likely disinclined to seek medical attention unless their health condition impedes their work productivity. This delay in seeking medical care may heighten the risk for moderate-to-severe COVID-19 development, consequently increasing the likelihood of subsequent sequelae. Given these circumstances, informal workers hospitalized due to COVID-19 may prioritize swift return to their labor activities over rest or medical appointments for identified neuropsychiatric sequelae.

### 4.2. Governmental pandemic control measures

This aspect closely interrelates with the former, as quarantine lockdowns and curfews substantially impacted the informal economy sector, accentuating the urgency to work for economic recovery. Moreover, governments opted for home confinement, while the reality is that many households lack access to potable water, and around 20% of Latin America’s population resides in precarious neighborhoods characterized by overcrowding where adherence to measures like social distancing is practically impossible (33).

### 4.3. Profound blow to the economic sector

It is evident that SARS-CoV-2 significantly impacted the global economy; however, according to the World Bank, Latin America suffered the greatest economic repercussions (34). In the first half of 2020 alone, the region lost 47 million jobs (32). The pandemic is estimated to have led to an increase in poverty by over 50%. Given that most informal workers lacked adequate savings to weather a seemingly unending pandemic, many opted to disregard lockdown measures and continue working, even at the risk of virus exposure. The economic crisis substantially contributed to the resource scarcity experienced by various healthcare centers.

### 4.4. Deficiencies in healthcare systems and access to medical care in SA

Initially, the region’s healthcare systems faced inadequate funding, manifesting as shortages of vital resources that compromised healthcare centers’ capacity for resolution. Broadly, healthcare in SA is fragmented into three subsystems: a basic and precarious one targeting low-income individuals, a more structured system catering to formal workers’ needs, and a third, typically private, system boasting better equipment and quality, reserved for the higher socioeconomic strata. This fragmentation hinders adequate access to healthcare for the population, with a substantial portion of informal workers predominantly accessing the most basic and under-equipped subsystem, devoid of access to the other two (35). Although all healthcare subsystems were susceptible to becoming overwhelmed during the pandemic, the most basic one suffered the most, resulting in prolonged appointment waiting times.

Numerous other factors likely contribute to the substantial impact of COVID-19 in SA, which, in turn, could significantly explain the heightened frequency of neuropsychiatric sequelae compared to other parts of the world. Undeniably, SA was declared the pandemic epicenter in 2020, accounting for over 40% of global COVID-19 fatalities in the same year (33). However, it is evident that high informality levels, lack of social protection, healthcare system fragmentation, and the severe economic setback were significant contributing factors to SA’s COVID-19 impact.

Nevertheless, the prevalence of neuropsychiatric disorders, such as sleep disorders, anxiety, depression, post-traumatic stress disorder, and cognitive impairment, were higher in our study compared to the rest of the world, as demonstrated by the meta-analysis conducted by Zeng et al. (12).

There is also a condition known as post-intensive care syndrome, which encompasses a series of cognitive, psychiatric, physical, and pulmonary disorders observed after a stay in the intensive care unit (ICU) across multiple pathologies (36). Proportions of around 30% of cognitive and psychiatric disorders have been reported in this population (37). A retrospective cohort of 280,000 patients in each group, using electronic health records from various countries, demonstrated that individuals hospitalized for COVID-19 had a higher risk of neurological and psychiatric sequelae compared to other pathologies; however, among patients who were in the ICU, no significant differences were found (38). We were unable to separately evaluate patients who were hospitalized but not admitted to the ICU and those who were, due to limited data availability in the reviewed literature. It would be useful for further studies to differentiate between these two groups to determine how much of the effect is attributed to long COVID or post-ICU syndrome.

There is great pressure on health care systems in SA due to COVID-19, but there are few studies on the impact of long-COVID (39). This could result in health authorities not prioritizing the creation and implementation of rehabilitation programs to help the affected population recover. Despite this, the prevalence of neuropsychiatric disorders, such as sleep disorders, anxiety, depression, PTSD, and cognitive impairment were higher in our study compared to the rest of the world according to the meta-analysis conducted by Zeng et al. (12). The population that is in the process of recovering may not seek assistance for their rehabilitation due to the prevalence of informal work arrangements and the urgency to return to work as soon as possible, given the socioeconomic context of the majority of people (33). Therefore, it is important that health authorities, healthcare personnel, and the community in general take measures to prevent these individuals from once again becoming major victims of COVID-19.

Three decades ago, as part of the Caracas Declaration, Latin American countries committed to the transformation of psychiatric care. This transformation involved shifting from the traditional model focused on psychiatric hospitals to a community-based approach centered on mental health facilities. Despite these intentions, in many Latin American countries, psychiatric hospitals continue to play a significant role in mental health care and operate within the traditional model. Consequently, it is imperative for countries to advance toward a transition to community-based mental health care. This transition entails the development and strengthening of mental health services within the community and a reduction in the number of beds in psychiatric institutions designated for extended stays. Achieving this transition will require sustained planning, resource allocation, and appropriate policies (40).

### 4.5. Sources of heterogeneity

We report a higher prevalence of insomnia and PTSD in studies conducted in countries such as Peru and Chile, as well as in cities at altitudes between 500 and 1,000 m. This is consistent with a Peruvian study of high-altitude cities in which a high prevalence of some neuropsychiatric disorders, such as PTSD and bipolar disorder, was found in patients with COVID-19 (41). In addition, an Ecuadorian study found a greater persistence of symptoms such as mood swings, insomnia, and decreased libido in high-altitude cities (>2,500 m) (42).

We found that a higher prevalence of neuropsychiatric disorders was reported in studies with a moderate risk of bias and those that were longitudinal. It has been shown that a high risk of bias in clinical trials can lead to overestimation of the treatment effect, which could be consistent with our results (43). In our case, some studies presented a risk of bias during the sampling process, and thus, the individual estimate of the studies could be compromised.

Despite the broad characterization of the studies included, the small sample size did not allow adequate evaluation of the causes of heterogeneity. However, in other reviews, it has been identified that variables, such as age, sex, working hours per week, and the medical profession, were associated with a higher prevalence of neuropsychiatric disorders (44, 45). For this reason, we recommend carrying out a meta-analysis with future publications that evaluate the long-term prevalence of neuropsychiatric disorders in SA.

### 4.6. Limitations

This systematic review has some limitations that need to be mentioned. Firstly, we have not conducted a search in gray literature. This could be relevant since some Latin American authors might publish in journals not indexed in the databases we have reviewed. Secondly, the paucity of studies conducted in SA has hindered comprehensive statistical analysis and therefore, proper evaluation of heterogeneity and an appropriate sensitivity analysis could not be performed. Thirdly, most of the studies included in the review relied on self-reported outcomes rather than structured clinical assessments. It is plausible that the extent and frequency of these symptoms may be part of the natural course of recovery from severe viral illness. Additionally, the time interval between hospitalization and data collection of the neuropsychiatric disorders varied among the studies included in the review. Fourthly, most of the studies did not follow-up the neuropsychiatric disorders in real-time; that is, they did not conduct a baseline measurement of the disorders before or during the acute infection, and then track changes over time; instead, they used a cross-sectional approach. This could also contribute to potential measurement bias. Finally, there is a dearth of studies in the post-hospitalized population, which is a high-risk group, precluding accurate comparisons from being established.

### 4.7. Conclusion

This review shows that the prevalence of neuropsychiatric disorders following hospitalization for COVID-19 in SA is higher compared to other regions, although these findings are merely exploratory. Neuropsychiatric disorders should be a priority for research and health care management in SA, and public policies should be implemented to help identify and treat these disorders in a timely manner to improve the quality of life of these patients. Additional research is necessary to enhance our comprehension of the prevalence of various neuropsychiatric symptoms linked with the long COVID-19 syndrome in South America. In order to achieve this objective, the implementation of standardized diagnostic methodologies is pivotal in enabling a comprehensive evaluation of symptom prevalence and their correlated factors among the South American population. This approach will enable significant cross-comparisons with studies conducted in different geographical settings.

## Data availability statement

The raw data supporting the conclusions of this article will be made available by the authors, without undue reservation.

## Author contributions

FP-F, NJ-M, AL-R, BG, VV-R, and CA-D: conceptualization. MC-L: search strategy. FP-F, NJ-M, AL-R, BG, MC-L, VV-R, and CA-D: selection. FP-F, NJ-M, AL-R, and BG: extraction. AL-R, BG, and MC-L: quality. MC-L, VV-R, and CA-D: analysis and data gathering, cleaning, and analysis. VV-R and CA-D: supervising processes. All authors contributed to the article and approved the submitted version.

## Footnotes

^1^https://rayyan.qcri.org/
