# Supplementary material for: Prevalence and incidence of neuropsychiatric disorders in post hospitalized COVID-19 patients in South America: a systematic review and meta-analysis
Source: Front Psychiatry. 2023 Nov 1;14:1163989. doi: 10.3389/fpsyt.2023.1163989 (PMC10646553; doi:10.3389/fpsyt.2023.1163989)
Supplement: Supplementary file 1 [file Table_1.DOCX]

Supplementary Material

**Prevalence and incidence of neuropsychiatric disorders in post-hospitalized COVID-19 patients in South America: A systematic review and meta-analysis**

**Francisco Perea-Flórez, Nair Javier-Murillo, André Lapeyre-Rivera, Bryan Gamonal, Miguel Cabanillas-Lazo, Víctor Velásquez-Rimachi, Carlos Alva-Díaz**

**Correspondence:**

**Carlos Alva-Díaz** (alvacarl@crece.uss.edu.pe)

**Víctor Velásquez-Rimachi**(vvelasquezr@cientifica.edu.pe)

# Search strategy

| **Scopus** | | |
| --- | --- | --- |
| Step | Search query | Results |
| #1 | TITLE-ABS(Inpatient* OR (Hospital* W/2 patient*) OR Hospitalised OR hospitalized OR Inhospital OR Hospitalizat* OR survivor* OR discharg* OR admiss* OR admit* OR intubat* OR icu OR ((Intensiv* OR close OR critical* OR respiratory OR special*) W/2 (care OR attention* OR therapy OR treatment*) W/2 (unit* OR depart*))) OR AUTHKEY(Inpatient* OR (Hospital* W/2 patient*) OR Hospitalised OR hospitalized OR Inhospital OR Hospitalizat* OR survivor* OR discharg* OR admiss* OR admit* OR intubat* OR icu OR ((Intensiv* OR close OR critical* OR respiratory OR special*) W/2 (care OR attention* OR therapy OR treatment*) W/2 (unit* OR depart*))) |  |
| #2 | TITLE-ABS(( 2019*cov OR ncov OR ( ( cov ) W/2 ( 19 OR 2019 OR 2 ) ) OR ( covid W/2 ( 19 OR 2019 OR 2 ) ) OR covid19 OR ( ( coronavirus OR "Corona virus" OR cov ) W/2 ( disease OR infection ) W/2 ( 2019 OR 19 OR 2 ) ) OR ( ( sars OR "Severe acute respiratory syndrome" OR sras ) W/2 ( cov OR coronavirus OR "Corona virus" OR covid ) W/2 ( "2" OR 2019 OR 19 ) ) OR "SARS-CoV2" OR sarscov2 OR "SRAS-CoV2" OR "Severe acute respiratory syndrome COV2" OR ( ( ( ( novel OR wuhan OR china OR pandemi* OR outbreak OR "new human" OR crisis OR "new cases" OR "normalcy" ) W/2 ( coronaviru* OR "corona viru*" OR covid ) ) OR ( "new corona*" AND NOT ( coronar* ) ) ) ) OR "Corona pandemic" OR ( wuhan W/2 pneumonia ) OR "Corona crisis" OR "Corona outbreak" )) OR AUTHKEY(( 2019*cov OR ncov OR ( ( cov ) W/2 ( 19 OR 2019 OR 2 ) ) OR ( covid W/2 ( 19 OR 2019 OR 2 ) ) OR covid19 OR ( ( coronavirus OR "Corona virus" OR cov ) W/2 ( disease OR infection ) W/2 ( 2019 OR 19 OR 2 ) ) OR ( ( sars OR "Severe acute respiratory syndrome" OR sras ) W/2 ( cov OR coronavirus OR "Corona virus" OR covid ) W/2 ( "2" OR 2019 OR 19 ) ) OR "SARS-CoV2" OR sarscov2 OR "SRAS-CoV2" OR "Severe acute respiratory syndrome COV2" OR ( ( ( ( novel OR wuhan OR china OR pandemi* OR outbreak OR "new human" OR crisis OR "new cases" OR "normalcy" ) W/2 ( coronaviru* OR "corona viru*" OR covid ) ) OR ( "new corona*" AND NOT ( coronar* ) ) ) ) OR "Corona pandemic" OR ( wuhan W/2 pneumonia ) OR "Corona crisis" OR "Corona outbreak" )) |  |
| #3 | TITLE-ABS-KEY(“Argentin*” OR “Aruba” OR “Bolivia*” OR “Brasil*” OR “Brazil*” OR “Chile*” OR “Colombia*” OR “Ecuador*” OR “French Guiana” OR “Guiana” OR “Guyana” OR “Paraguay*” OR “Peru*” OR “Suriname” OR “Surinam” OR “Trinidad and Tobago” OR “Uruguay*” OR “Venezuela*” OR “South America*”) |  |
|  | (#1 AND #2 AND #3) | 1363 |

| **PubMed/Medline** | | |
| --- | --- | --- |
| Step | Search query | Results |
| #1 | Inpatients[MH] OR Hospitalization[mh] OR Inpatient*[tiab] OR Patient Admission[MH] OR Patient Discharge[MH] OR “Hospital patient*”[tiab] OR Hospitalised[tiab] OR hospitalized[tiab] OR Inhospital[tiab] OR Hospitalizat*[tiab] OR survivor*[tiab] OR discharg*[tiab] OR admiss*[tiab] OR admit*[tiab] OR Intensive Care Units[MH] OR “Intensive Care Unit*”[tiab] OR “close attention unit*”[tiab] OR “critical care unit*”[tiab] OR “intensive care depart*”[tiab] OR “intensive therapy unit*”[tiab] OR “intensive treatment unit*”[tiab] OR “respiratory care unit*”[tiab] OR “special care unit*”[tiab] OR ICU[tiab] OR Intubation, Intratracheal[MH] OR intubat*[tiab] |  |
| #2 | COVID-19[MH] OR SARS-CoV-2[MH] OR post-acute COVID-19 syndrome [Supplementary Concept] OR Ncov[tiab] OR “COVID 19”[tiab] OR covid19[tiab] OR “covid 2019”[tiab] OR covid2019[tiab] OR “covid 2”[tiab] OR covid2[tiab] OR “COV 19”[tiab] OR cov19[tiab] OR “cov 2019”[tiab] OR cov2019[tiab] OR “cov 2”[tiab] OR cov2[tiab] OR ((coronavirus[tiab] OR “corona virus” [tiab] OR cov[tiab]) AND (disease[tiab] OR infection[tiab]) AND (2019[tiab] OR 19[tiab] OR 2[tiab])) OR ((novel[tiab] OR wuhan[tiab] OR china[tiab] OR pandemi*[tiab] OR outbreak[tiab] OR "new human"[tiab] OR crisis[tiab] OR "new cases"[tiab] OR “normalcy"[tiab]) AND (coronaviru*[tiab] OR "corona viru*"[tiab] OR covid[tiab])) OR "Corona pandemic"[tiab] OR (wuhan[tiab] AND pneumonia[tiab]) OR “Long COVID*”[tiab] OR “Long haul COVID*”[tiab] OR “COVID 19 long*”[tiab] OR “post covid*”[tiab] OR “post acute COVID*”[tiab] OR “persistent COVID*”[tiab] OR “chronic COVID*”[tiab] OR (((Sequelae[tiab] OR “postacute”[tiab] OR “post acute”[tiab]) AND (covid*[tiab] OR cov2[tiab] OR “cov 2”[tiab])) OR ((Follow-Up Studies[MH] OR Followup[tiab] OR “follow up”[tiab]) AND (covid*[tiab] OR cov2[tiab] OR “cov 2”[tiab]))) |  |
| #3 | South America[MH] OR Argentina[MH] OR Bolivia[MH] OR Brazil[MH] OR Chile[MH] OR Colombia[MH] OR Ecuador[MH] OR French Guiana[MH] OR Guyana[MH] OR Paraguay[MH] OR Peru[MH] OR Suriname[MH] OR Uruguay[MH] OR Venezuela[MH] OR (“Argentin*”[tiab] OR “Aruba”[tiab] OR “Bolivia*”[tiab] OR “Brasil*”[tiab] OR “Brazil*”[tiab] OR “Chile*”[tiab] OR “Colombia*”[tiab] OR “Ecuador*”[tiab] OR “French Guiana”[tiab] OR “Guiana”[tiab] OR “Guyana”[tiab] OR “Paraguay*”[tiab] OR “Peru*”[tiab] OR “Suriname”[tiab] OR “Surinam”[tiab] OR “Trinidad and Tobago”[tiab] OR “Uruguay*”[tiab] OR “Venezuela*”[tiab] OR “South America*”[tiab]) |  |
| #5 | (#1 AND #2 AND #3) | 1224 |

| **Embase** | | |
| --- | --- | --- |
| Step | Search query | Results |
| #A | #1: (Inpatient* OR (Hospital* NEAR/2 patient*) OR Hospitalised OR hospitalized OR Inhospital OR Hospitalizat* OR survivor* OR discharg* OR admiss* OR admit* OR intubat* OR icu OR ((Intensiv* OR close OR critical* OR respiratory OR special*) NEAR/2 (care OR attention* OR therapy OR treatment*) NEAR/2 (unit* OR depart*))):ti,ab,kw  #2: 'hospital patient'/exp OR 'hospitalization'/exp OR 'hospital admission'/exp OR 'hospital discharge'/exp |  |
| #B | #1: (( 2019*cov OR ncov OR ( ( cov ) NEAR/2 ( 19 OR 2019 OR 2 ) ) OR ( covid NEAR/2 ( 19 OR 2019 OR 2 ) ) OR covid19 OR ( ( coronavirus OR "Corona virus" OR cov ) NEAR/2 ( disease OR infection ) NEAR/2 ( 2019 OR 19 OR 2 ) ) OR ( ( sars OR "Severe acute respiratory syndrome" OR sras ) NEAR/2 ( cov OR coronavirus OR "Corona virus" OR covid ) NEAR/2 ( "2" OR 2019 OR 19 ) ) OR "SARS-CoV2" OR sarscov2 OR "SRAS-CoV2" OR "Severe acute respiratory syndrome COV2" OR ( ( ( ( novel OR wuhan OR china OR pandemi* OR outbreak OR "new human" OR crisis OR "new cases" OR "normalcy" ) NEAR/2 ( coronaviru* OR "corona viru*" OR covid ) ) OR ( "new corona*" NOT ( coronar* ) ) ) ) OR "Corona pandemic" OR ( wuhan NEAR/2 pneumonia ) OR "Corona crisis" OR "Corona outbreak" )):ti,ab,kw  #2: 'coronavirus disease 2019'/exp  #3: 'Severe acute respiratory syndrome coronavirus 2'/exp |  |
| #C | #1: (“Argentin*” OR “Aruba” OR “Bolivia*” OR “Brasil*” OR “Brazil*” OR “Chile*” OR “Colombia*” OR “Ecuador*” OR “French Guiana” OR “Guiana” OR “Guyana” OR “Paraguay*” OR “Peru*” OR “Suriname” OR “Surinam” OR “Trinidad and Tobago” OR “Uruguay*” OR “Venezuela*” OR “South America*”):ti,ab,kw  #2: 'Argentina'/exp OR 'Argentinian'/exp OR 'Aruba'/exp OR 'Bolivia'/exp OR 'Bolivian'/exp OR 'Brazil'/exp OR 'Brazilian'/exp OR 'Chile'/exp OR 'Chilean'/exp OR 'Colombia'/exp OR 'Colombian'/exp OR 'Ecuador'/exp OR 'Ecuadorean'/exp OR 'French Guiana'/exp OR 'Guyana'/exp OR 'Guyanese'/exp OR 'Paraguay'/exp OR 'Paraguayan'/exp OR 'Peru'/exp OR 'Peruvian'/exp OR 'Suriname'/exp OR 'Surinamese'/exp OR 'Trinidad and Tobago'/exp OR 'Uruguay'/exp OR 'Uruguayan'/exp OR 'Venezuela'/exp OR 'Venezuelan'/exp OR 'South America'/exp OR 'South american'/exp  1749 |  |
| #D | #A AND #B AND #C 1749 |  |

| **Web of Science** | | |
| --- | --- | --- |
| Step | Search query | Results |
| #1 | TI=(Inpatient* OR (Hospital* NEAR/2 patient*) OR Hospitalised OR hospitalized OR Inhospital OR Hospitalizat* OR survivor* OR discharg* OR admiss* OR admit* OR intubat* OR icu OR ((Intensiv* OR close OR critical* OR respiratory OR special*) NEAR/2 (care OR attention* OR therapy OR treatment*) NEAR/2 (unit* OR depart*))) OR AB=(Inpatient* OR (Hospital* NEAR/2 patient*) OR Hospitalised OR hospitalized OR Inhospital OR Hospitalizat* OR survivor* OR discharg* OR admiss* OR admit* OR intubat* OR icu OR ((Intensiv* OR close OR critical* OR respiratory OR special*) NEAR/2 (care OR attention* OR therapy OR treatment*) NEAR/2 (unit* OR depart*))) OR AK=(Inpatient* OR (Hospital* NEAR/2 patient*) OR Hospitalised OR hospitalized OR Inhospital OR Hospitalizat* OR survivor* OR discharg* OR admiss* OR admit* OR intubat* OR icu OR ((Intensiv* OR close OR critical* OR respiratory OR special*) NEAR/2 (care OR attention* OR therapy OR treatment*) NEAR/2 (unit* OR depart*))) OR KP=(Inpatient* OR (Hospital* NEAR/2 patient*) OR Hospitalised OR hospitalized OR Inhospital OR Hospitalizat* OR survivor* OR discharg* OR admiss* OR admit* OR intubat* OR icu OR ((Intensiv* OR close OR critical* OR respiratory OR special*) NEAR/2 (care OR attention* OR therapy OR treatment*) NEAR/2 (unit* OR depart*))) OR TS=(Inpatient* OR (Hospital* NEAR/2 patient*) OR Hospitalised OR hospitalized OR Inhospital OR Hospitalizat* OR survivor* OR discharg* OR admiss* OR admit* OR intubat* OR icu OR ((Intensiv* OR close OR critical* OR respiratory OR special*) NEAR/2 (care OR attention* OR therapy OR treatment*) NEAR/2 (unit* OR depart*))) |  |
| #2 | TI=(( 2019*cov OR ncov OR ( ( cov ) NEAR/2 ( 19 OR 2019 OR 2 ) ) OR ( covid NEAR/2 ( 19 OR 2019 OR 2 ) ) OR covid19 OR ( ( coronavirus OR "Corona virus" OR cov ) NEAR/2 ( disease OR infection ) NEAR/2 ( 2019 OR 19 OR 2 ) ) OR ( ( sars OR "Severe acute respiratory syndrome" OR sras ) NEAR/2 ( cov OR coronavirus OR "Corona virus" OR covid ) NEAR/2 ( "2" OR 2019 OR 19 ) ) OR "SARS-CoV2" OR sarscov2 OR "SRAS-CoV2" OR "Severe acute respiratory syndrome COV2" OR ( ( ( ( novel OR wuhan OR china OR pandemi* OR outbreak OR "new human" OR crisis OR "new cases" OR "normalcy" ) NEAR/2 ( coronaviru* OR "corona viru*" OR covid ) ) OR ( "new corona*" NOT ( coronar* ) ) ) ) OR "Corona pandemic" OR ( wuhan NEAR/2 pneumonia ) OR "Corona crisis" OR "Corona outbreak" )) OR AB=(( 2019*cov OR ncov OR ( ( cov ) NEAR/2 ( 19 OR 2019 OR 2 ) ) OR ( covid NEAR/2 ( 19 OR 2019 OR 2 ) ) OR covid19 OR ( ( coronavirus OR "Corona virus" OR cov ) NEAR/2 ( disease OR infection ) NEAR/2 ( 2019 OR 19 OR 2 ) ) OR ( ( sars OR "Severe acute respiratory syndrome" OR sras ) NEAR/2 ( cov OR coronavirus OR "Corona virus" OR covid ) NEAR/2 ( "2" OR 2019 OR 19 ) ) OR "SARS-CoV2" OR sarscov2 OR "SRAS-CoV2" OR "Severe acute respiratory syndrome COV2" OR ( ( ( ( novel OR wuhan OR china OR pandemi* OR outbreak OR "new human" OR crisis OR "new cases" OR "normalcy" ) NEAR/2 ( coronaviru* OR "corona viru*" OR covid ) ) OR ( "new corona*" NOT ( coronar* ) ) ) ) OR "Corona pandemic" OR ( wuhan NEAR/2 pneumonia ) OR "Corona crisis" OR "Corona outbreak" )) OR AK=(( 2019*cov OR ncov OR ( ( cov ) NEAR/2 ( 19 OR 2019 OR 2 ) ) OR ( covid NEAR/2 ( 19 OR 2019 OR 2 ) ) OR covid19 OR ( ( coronavirus OR "Corona virus" OR cov ) NEAR/2 ( disease OR infection ) NEAR/2 ( 2019 OR 19 OR 2 ) ) OR ( ( sars OR "Severe acute respiratory syndrome" OR sras ) NEAR/2 ( cov OR coronavirus OR "Corona virus" OR covid ) NEAR/2 ( "2" OR 2019 OR 19 ) ) OR "SARS-CoV2" OR sarscov2 OR "SRAS-CoV2" OR "Severe acute respiratory syndrome COV2" OR ( ( ( ( novel OR wuhan OR china OR pandemi* OR outbreak OR "new human" OR crisis OR "new cases" OR "normalcy" ) NEAR/2 ( coronaviru* OR "corona viru*" OR covid ) ) OR ( "new corona*" NOT ( coronar* ) ) ) ) OR "Corona pandemic" OR ( wuhan NEAR/2 pneumonia ) OR "Corona crisis" OR "Corona outbreak" )) OR KP=(( 2019*cov OR ncov OR ( ( cov ) NEAR/2 ( 19 OR 2019 OR 2 ) ) OR ( covid NEAR/2 ( 19 OR 2019 OR 2 ) ) OR covid19 OR ( ( coronavirus OR "Corona virus" OR cov ) NEAR/2 ( disease OR infection ) NEAR/2 ( 2019 OR 19 OR 2 ) ) OR ( ( sars OR "Severe acute respiratory syndrome" OR sras ) NEAR/2 ( cov OR coronavirus OR "Corona virus" OR covid ) NEAR/2 ( "2" OR 2019 OR 19 ) ) OR "SARS-CoV2" OR sarscov2 OR "SRAS-CoV2" OR "Severe acute respiratory syndrome COV2" OR ( ( ( ( novel OR wuhan OR china OR pandemi* OR outbreak OR "new human" OR crisis OR "new cases" OR "normalcy" ) NEAR/2 ( coronaviru* OR "corona viru*" OR covid ) ) OR ( "new corona*" NOT ( coronar* ) ) ) ) OR "Corona pandemic" OR ( wuhan NEAR/2 pneumonia ) OR "Corona crisis" OR "Corona outbreak" )) OR TS=(( 2019*cov OR ncov OR ( ( cov ) NEAR/2 ( 19 OR 2019 OR 2 ) ) OR ( covid NEAR/2 ( 19 OR 2019 OR 2 ) ) OR covid19 OR ( ( coronavirus OR "Corona virus" OR cov ) NEAR/2 ( disease OR infection ) NEAR/2 ( 2019 OR 19 OR 2 ) ) OR ( ( sars OR "Severe acute respiratory syndrome" OR sras ) NEAR/2 ( cov OR coronavirus OR "Corona virus" OR covid ) NEAR/2 ( "2" OR 2019 OR 19 ) ) OR "SARS-CoV2" OR sarscov2 OR "SRAS-CoV2" OR "Severe acute respiratory syndrome COV2" OR ( ( ( ( novel OR wuhan OR china OR pandemi* OR outbreak OR "new human" OR crisis OR "new cases" OR "normalcy" ) NEAR/2 ( coronaviru* OR "corona viru*" OR covid ) ) OR ( "new corona*" NOT ( coronar* ) ) ) ) OR "Corona pandemic" OR ( wuhan NEAR/2 pneumonia ) OR "Corona crisis" OR "Corona outbreak" )) |  |
| #3 | TI=(“Argentin*” OR “Aruba” OR “Bolivia*” OR “Brasil*” OR “Brazil*” OR “Chile*” OR “Colombia*” OR “Ecuador*” OR “French Guiana” OR “Guiana” OR “Guyana” OR “Paraguay*” OR “Peru*” OR “Suriname” OR “Surinam” OR “Trinidad and Tobago” OR “Uruguay*” OR “Venezuela*” OR “South America*”) OR AB=(“Argentin*” OR “Aruba” OR “Bolivia*” OR “Brasil*” OR “Brazil*” OR “Chile*” OR “Colombia*” OR “Ecuador*” OR “French Guiana” OR “Guiana” OR “Guyana” OR “Paraguay*” OR “Peru*” OR “Suriname” OR “Surinam” OR “Trinidad and Tobago” OR “Uruguay*” OR “Venezuela*” OR “South America*”) OR AK=(“Argentin*” OR “Aruba” OR “Bolivia*” OR “Brasil*” OR “Brazil*” OR “Chile*” OR “Colombia*” OR “Ecuador*” OR “French Guiana” OR “Guiana” OR “Guyana” OR “Paraguay*” OR “Peru*” OR “Suriname” OR “Surinam” OR “Trinidad and Tobago” OR “Uruguay*” OR “Venezuela*” OR “South America*”) OR KP=(“Argentin*” OR “Aruba” OR “Bolivia*” OR “Brasil*” OR “Brazil*” OR “Chile*” OR “Colombia*” OR “Ecuador*” OR “French Guiana” OR “Guiana” OR “Guyana” OR “Paraguay*” OR “Peru*” OR “Suriname” OR “Surinam” OR “Trinidad and Tobago” OR “Uruguay*” OR “Venezuela*” OR “South America*”) OR TS=(“Argentin*” OR “Aruba” OR “Bolivia*” OR “Brasil*” OR “Brazil*” OR “Chile*” OR “Colombia*” OR “Ecuador*” OR “French Guiana” OR “Guiana” OR “Guyana” OR “Paraguay*” OR “Peru*” OR “Suriname” OR “Surinam” OR “Trinidad and Tobago” OR “Uruguay*” OR “Venezuela*” OR “South America*”)  822 |  |
| #3 | ((#1 AND #2 AND #3) 822 |  |

| **SCIELO (Web of Science)** | | |
| --- | --- | --- |
| Step | Search query | Results |
| #1 | TI=(Inpatient* OR (Hospital* NEAR/2 patient*) OR Hospitalised OR hospitalized OR Inhospital OR Hospitalizat* OR survivor* OR discharg* OR admiss* OR admit* OR intubat* OR icu OR ((Intensiv* OR close OR critical* OR respiratory OR special*) NEAR/2 (care OR attention* OR therapy OR treatment*) NEAR/2 (unit* OR depart*))) OR AB=(Inpatient* OR (Hospital* NEAR/2 patient*) OR Hospitalised OR hospitalized OR Inhospital OR Hospitalizat* OR survivor* OR discharg* OR admiss* OR admit* OR intubat* OR icu OR ((Intensiv* OR close OR critical* OR respiratory OR special*) NEAR/2 (care OR attention* OR therapy OR treatment*) NEAR/2 (unit* OR depart*))) OR AK=(Inpatient* OR (Hospital* NEAR/2 patient*) OR Hospitalised OR hospitalized OR Inhospital OR Hospitalizat* OR survivor* OR discharg* OR admiss* OR admit* OR intubat* OR icu OR ((Intensiv* OR close OR critical* OR respiratory OR special*) NEAR/2 (care OR attention* OR therapy OR treatment*) NEAR/2 (unit* OR depart*))) OR TS=(Inpatient* OR (Hospital* NEAR/2 patient*) OR Hospitalised OR hospitalized OR Inhospital OR Hospitalizat* OR survivor* OR discharg* OR admiss* OR admit* OR intubat* OR icu OR ((Intensiv* OR close OR critical* OR respiratory OR special*) NEAR/2 (care OR attention* OR therapy OR treatment*) NEAR/2 (unit* OR depart*))) |  |
| #2 | TI=(( 2019*cov OR ncov OR ( ( cov ) NEAR/2 ( 19 OR 2019 OR 2 ) ) OR ( covid NEAR/2 ( 19 OR 2019 OR 2 ) ) OR covid19 OR ( ( coronavirus OR "Corona virus" OR cov ) NEAR/2 ( disease OR infection ) NEAR/2 ( 2019 OR 19 OR 2 ) ) OR ( ( sars OR "Severe acute respiratory syndrome" OR sras ) NEAR/2 ( cov OR coronavirus OR "Corona virus" OR covid ) NEAR/2 ( "2" OR 2019 OR 19 ) ) OR "SARS-CoV2" OR sarscov2 OR "SRAS-CoV2" OR "Severe acute respiratory syndrome COV2" OR ( ( ( ( novel OR wuhan OR china OR pandemi* OR outbreak OR "new human" OR crisis OR "new cases" OR "normalcy" ) NEAR/2 ( coronaviru* OR "corona viru*" OR covid ) ) OR ( "new corona*" NOT ( coronar* ) ) ) ) OR "Corona pandemic" OR ( wuhan NEAR/2 pneumonia ) OR "Corona crisis" OR "Corona outbreak" )) OR AB=(( 2019*cov OR ncov OR ( ( cov ) NEAR/2 ( 19 OR 2019 OR 2 ) ) OR ( covid NEAR/2 ( 19 OR 2019 OR 2 ) ) OR covid19 OR ( ( coronavirus OR "Corona virus" OR cov ) NEAR/2 ( disease OR infection ) NEAR/2 ( 2019 OR 19 OR 2 ) ) OR ( ( sars OR "Severe acute respiratory syndrome" OR sras ) NEAR/2 ( cov OR coronavirus OR "Corona virus" OR covid ) NEAR/2 ( "2" OR 2019 OR 19 ) ) OR "SARS-CoV2" OR sarscov2 OR "SRAS-CoV2" OR "Severe acute respiratory syndrome COV2" OR ( ( ( ( novel OR wuhan OR china OR pandemi* OR outbreak OR "new human" OR crisis OR "new cases" OR "normalcy" ) NEAR/2 ( coronaviru* OR "corona viru*" OR covid ) ) OR ( "new corona*" NOT ( coronar* ) ) ) ) OR "Corona pandemic" OR ( wuhan NEAR/2 pneumonia ) OR "Corona crisis" OR "Corona outbreak" )) OR AK=(( 2019*cov OR ncov OR ( ( cov ) NEAR/2 ( 19 OR 2019 OR 2 ) ) OR ( covid NEAR/2 ( 19 OR 2019 OR 2 ) ) OR covid19 OR ( ( coronavirus OR "Corona virus" OR cov ) NEAR/2 ( disease OR infection ) NEAR/2 ( 2019 OR 19 OR 2 ) ) OR ( ( sars OR "Severe acute respiratory syndrome" OR sras ) NEAR/2 ( cov OR coronavirus OR "Corona virus" OR covid ) NEAR/2 ( "2" OR 2019 OR 19 ) ) OR "SARS-CoV2" OR sarscov2 OR "SRAS-CoV2" OR "Severe acute respiratory syndrome COV2" OR ( ( ( ( novel OR wuhan OR china OR pandemi* OR outbreak OR "new human" OR crisis OR "new cases" OR "normalcy" ) NEAR/2 ( coronaviru* OR "corona viru*" OR covid ) ) OR ( "new corona*" NOT ( coronar* ) ) ) ) OR "Corona pandemic" OR ( wuhan NEAR/2 pneumonia ) OR "Corona crisis" OR "Corona outbreak" )) OR TS=(( 2019*cov OR ncov OR ( ( cov ) NEAR/2 ( 19 OR 2019 OR 2 ) ) OR ( covid NEAR/2 ( 19 OR 2019 OR 2 ) ) OR covid19 OR ( ( coronavirus OR "Corona virus" OR cov ) NEAR/2 ( disease OR infection ) NEAR/2 ( 2019 OR 19 OR 2 ) ) OR ( ( sars OR "Severe acute respiratory syndrome" OR sras ) NEAR/2 ( cov OR coronavirus OR "Corona virus" OR covid ) NEAR/2 ( "2" OR 2019 OR 19 ) ) OR "SARS-CoV2" OR sarscov2 OR "SRAS-CoV2" OR "Severe acute respiratory syndrome COV2" OR ( ( ( ( novel OR wuhan OR china OR pandemi* OR outbreak OR "new human" OR crisis OR "new cases" OR "normalcy" ) NEAR/2 ( coronaviru* OR "corona viru*" OR covid ) ) OR ( "new corona*" NOT ( coronar* ) ) ) ) OR "Corona pandemic" OR ( wuhan NEAR/2 pneumonia ) OR "Corona crisis" OR "Corona outbreak" ))  321 |  |
| #3 | #1 AND #2 |  |

| **GOOGLE SCHOLAR** | | |
| --- | --- | --- |
| Step | Search query | Results |
| #A | (”SARS-CoV-2” OR COVID*) AND (hospitaliza* OR sobreviv* OR supervivient* OR admitid* OR admision* OR internado* OR UCI) | 150 |

| **LILACS** | | |
| --- | --- | --- |
| Step | Search query | Results |
| #A | (”SARS-CoV-2” OR COVID*) AND (inpatient* OR hospitaliza* OR hospitalis* OR inhospital* OR survivor* OR sobreviv* OR supervivient* OR discharg* OR admiss* OR admit* OR admitid* OR admision* OR internado* OR icu OR UCI) | 1499 |

**
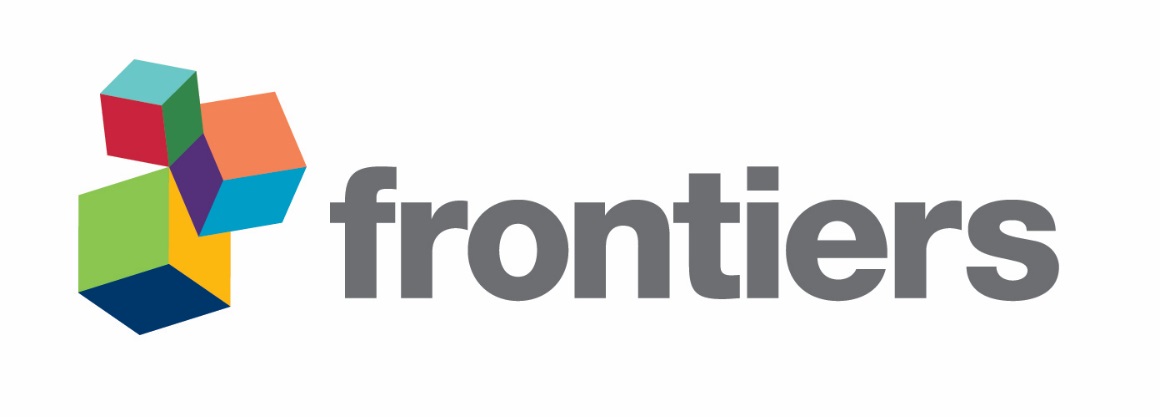
**
